# Adaptation to the driving simulator and prediction of the braking time performance, with and without distraction, in older adults and middle-aged adults

**DOI:** 10.1016/j.clinsp.2023.100168

**Published:** 2023-02-10

**Authors:** Alexandra Carolina Canonica, Angelica Castilho Alonso, Guilherme Carlos Brech, Mark Peterson, Natália Mariana Silva Luna, Alexandre Leopold Busse, Wilson Jacob-Filho, Juliana Leme Rosa, Jose Maria Soares-Junior, Edmund Chada Baracat, Júlia Maria D'Andrea Greve

**Affiliations:** a Laboratory Study of Movement, Instituto de Ortopedia e Traumatologia do Hospital das Clínicas (IOT-HC) da Faculdade de Medicina da Universidade de São Paulo (FMUSP), São Paulo, SP, Brazil; b Graduate Program in Aging Sciences, Universidade São Judas Tadeu (USJT), São Paulo, SP, Brazil; c Department of Physical Medicine and Rehabilitation, University of Michigan-Medicine, Michigan, United States; d Departamento de Geriatria da Faculdade de Medicina da Universidade de São Paulo (FMUSP), São Paulo, SP, Brazil; e Disciplina de Ginecologia, Departamento de Obstetrícia e Ginecologia, Hospital das Clínicas, Faculdade de Medicina da Universidade de São Paulo (FMUSP), São Paulo, SP, Brazil

**Keywords:** Drivers, Elderly, Cognitive distractor, Sex

## Abstract

**Context:**

Many studies show the importance of evaluating the adaptation time of subjects in a virtual driving environment, looking forwards to a response as closest as a possible real vehicle.

**Objectives:**

This study aimed to identify and analyze the adaptation to the driving simulator in older adults and middle-aged adults with and without a distraction, and a secondary aim was to identify predictors of safe performance for older adults' drives.

**Design:**

Male and female middle-aged adults (*n* = 62, age = 30.3 ± 7.1 years) and older adults (*n* = 102, age = 70.4 ± 5.8 years) were evaluated for braking time performance in a driving simulator; cognition performance assessment included the Mini-Mental State Examination; motor evaluation included ankle flexor muscle strength with the isokinetic dynamometer and handgrip strength; the postural balance was evaluated with Timed Up and Go test, with and without a cognitive distraction task.

**Results:**

Older adults (men and women) and middle-aged adult women require more time to adapt to the driving simulator. The distractor increases the adaptation time for all groups. The main predictors of braking time for older women are age, muscle strength, and postural balance associated with distraction, and for older men, muscle strength.

**Conclusions:**

Age, sex, and distractor interfere in the adaptation of the virtual task of driving in a simulator. The evaluation model developed with multi-domains demonstrated the ability to predict which skills are related to braking time with and without the presence of the distractor.

## Highlights

• What does interfere with the virtual task of driving?
• Older adults require a longer adaptation time to the driving simulator.
• Main predictors of braking time are age e muscle strength.

## Introduction

Driving is a task that involves a fast and dynamic interaction of hearing, vision, cognition, and psychomotor skills, which may be compromised by the aging process and lead to an increased risk of accidents.^1^ According to the Insurance Information Institute.^2^, in the United States, older drivers are the second largest group of individuals involved in fatal accidents.

In traffic, subjects need to keep their attention steady and divided to make correct decisions, but cognitive and auditory stimuli within (radio, mobile phones, and conversations) or outside of the vehicle (horns, headlights, and pedestrians) can be distracting and hinder driving. The mobile phone has been evaluated as an important factor of distraction, and a potential cause of accidents.^3^, ^4^, ^5^, ^6^

Many studies evaluated the effect of distractors during the driving task in the driving simulator. There is an adaptation period for the use of the driving simulator (virtual environment) before the evaluation, excluding the learning effect of the equipment, for the closest result to that obtained in driving a real vehicle.^7^ There are only a few studies on the adaptation time (motor and cognitive) for the use of the driving simulator, especially in older adults.^7^

Thus, this study aimed to identify and analyze the adaptation to the driving simulator in older adults and middle-aged adults with and without a distraction; and the secondary aim was to identify predictors of the safe performance of older adult drivers.

## Methods

### Experimental design, local and ethics

This was a cross-sectional study conducted was performed at the Motion Study Laboratory of the Institute of Orthopedics and Traumatology, Hospital das Clínicas, University of São Paulo School of Medicine, approved by the Ethics Research Committee (protocol number: 0468/10).

### Participants

The authors evaluated 164 males and females divided into two groups: 102 individuals over 65 (70.4 ± 5.8 years), recruited at the Geriatrics Outpatient Department of Hospital das Clinics, School of Medicine, University of São Paulo; and 62 middle-aged healthy adults between the ages of 30 and 40 (39.3 ± 7.1 years). The inclusion criteria were: to have a valid driver's license during the past five years; regular driver (at least two days per week); the absence of vestibular, proprioceptive, auditory, neurological, and/or mental impairment; not using any medication that could affect the ability to drive; the absence of diseases or surgeries in the lower limb, surgeries that could influence the mobility; without any functional limitations regarding the joint range of motion from the ankle, knee, and hip. Subjects who, for any reason, were unable to carry out one or more of the proposed evaluations were excluded from the study.

### Procedures

All subjects agreed to participate in the study by reading and signing the informed consent form. After that, they answered a questionnaire with personal information, socio-demographics, and driving history.

### Assessments

#### Cognitive assessments

Mini-Mental State Examination (MMSE) consists of 11 items (30-point) that assess domains of orientation, short-term memory, attention, and visual space.^8^

#### Motor assessments

Timed Up and Go Test (TUG)- Comprised of mobility, transfers, gait, agility, strength, and postural balance that are measured in TUG. Function test was performed with and without distractors as a cognitive task. The subjects performed the task of standing up from a chair, walking with a usual speed for ten feet, turning back, and sitting again. Time (in seconds) was measured. The TUG with distraction was performed with the same test adding the task of verbalizing animals' names.^9^

Isokinetic Dynamometry: Maximal dynamic strength of plantar flexor muscles of the dominant and non-dominant limb was measured using the isokinetic dynamometer (Biodex System 2, USA). Subjects were placed in a seated position with support in the distal region of the thigh and the sol resting on a rigid plate. The axis of the ankle joint was aligned with the mechanical axis of the dynamometer with the knee at 30° of flexion. Subjects were held in position with one pelvic and two thoracic belts, and velcro bands over the distal portion of the thigh and the area of the metatarsals in the dorsal region of the foot. Three submaximal attempts were performed to become familiarized with the equipment procedure. Verbal encouragement was given throughout the trials to motivate the subjects. Two tests were performed with five repetitions at the angular velocity of 30°/s, starting with the dominant limb. For the data analysis, the measurements of the second test (10) were considered. The following variables were used: Peak Torque corrected for Body Weight (PT/BW) given in percentage (%), and Total Work performed on the five repetitions (TW) given in Joules (J).^10^^,^^11^

Hand Grip strength: The Jamar® dynamometer was used. The subjects remained seated with their arms parallel to the body, shoulder adducted, elbow flexed at 90°, and forearm and wrist in a neutral position. Three measures were performed in the dominant and non-dominant hands in an intercalated fashion, with an interval of one minute between trials. For analysis, the average of the three values obtained in kilogram-force (kg/f) was used.^11^

#### Driving simulator test

The authors used the FOERST brand simulator, Car-Simulator “Trainer” Type F12PT. The specific task of driving was applied on a highway with single lanes and without traffic. The parameter used was the braking time. Subjects were instructed to sit in the simulator, adjust the seat and seatbelt and start the equipment with a key, similar to that used in real vehicles. The “reaction time” test was used, in which the word “stop” appeared randomly during the course and would indicate that subjects should brake. The braking time (in seconds) was collected between the appearance of the “stop” command and the subject pressing the brake with the right foot. This command was repeated five times and at the end of the 3.3 km course, five measurements of the braking times and the time taken to complete the course were collected.^11^ The procedure was repeated with the driver holding a conversation (as a distractor) with the evaluator about family and home aspects.

### Statistical analysis

The data was analyzed in SPSS Statistics 22.0 for Windows (SPSS, Inc.). Descriptive data were presented by means and standard deviations. The 5% level of significance was used in the statistical analysis. The normality and homogeneity of variances were confirmed by the Kolmogorov-Smirnov and Levene tests, respectively.

To observe the effect and interaction (repetitions * age * sex * distractor) between the groups, the authors used the analysis of variance with repeated measurements and the Bonferroni test with the post-hoc for multiple comparisons.

Simple linear regression (forward mode) was performed to investigate whether independent variables predict braking performance, separated by sex, and with or without the distractor with the older adults. The variables were included in the following order: Model 1: Sociodemographic; Model 2: Cognitive and sociodemographic, and Model 3: Sociodemographic, cognitive, and motor. The multiple linear regression model was used to associate the independent variables with braking time, separated by sex and with or without the distractor. Only the variables that showed association with *p* ≤ 0.05 were included in the final model. These variables were ranked from the lowest to the highest p-value. Independent variables were added to the model using stepwise forward selection (for example: variables were added one by one according to their position in the sequence). Only the independent variables with *p* ≤ 0.05 remained in the final model.

## Results

The baseline analysis is presented in Table 1.

**Table 1:** Demographic characteristics of the older adults and Middle-aged adult group.

	Older adults			Middle-aged adults		
	Women (*n* = 51)	Men (*n* = 51)	Total (*n* = 102)	Women (n=31)	Men (*n* = 31)	Total (*n* = 62)
	M (SD)	M (SD)	M (SD)	M (SD)	M (SD)	M (SD)
Age (years)	68.0 (4.5)	72.5 (5.7)	70.4 (5.8)	41.3 (6.9)	38.0 (6.9)	39.3 (7.1)
Years licensed	38.8 (7.7)	47.0 (8.9)	42.8 (9.2)	18.0 (6.4)	17.8 (7.3)	17.9 (6.6)
Years of education	12.5 (2.8)	12.5 (3.3)	12.6 (3.0)	16.1 (3.3)	15.3 (1.5)	15.7 (2.5)
MMSE	27.5(2.2)	27.5 (2.4)	27.5 (2.3)	28.1 (1.6)	28.3 (2.0)	28.3 (1.8)
Hand grip (kgf) (DS)	26.1 (5.3)	40.1 (9.0)	33.2(10.2)	29.6 (6.1)	49.7 (7.0)	39.7 (12.0)
Hand grip (kgf) (NDS)	23.7 (3.8)	36.3 (8.3)	30.1 (9.0)	27.0 (4.6)	47.1 (8.4)	37.0 (12.1)
PT/BW (%)	64.2 (23.6)	82.9 (34.4)	73.5 (30.8)	87.2 (34.4)	111.4 (38.2)	99.5 (38.1)
Total work (J)	60.3 (41.4)	96.5 (54.5)	78.4 (51.5)	85.7 (39.0)	139.4 (68.0)	113.0 (61.5)
TUGT (s)	7.6 (2.0)	7.7 (2.3)	7.6 (2.1)	6.1 (1.7)	5.7 (0.9)	5.9 (1.4)
Cognitive TUGT (s)	8.9 (3.3)	8.8 (2.9)	8.9 (3.1)	6.5 (2.0)	5.7 (0.9)	6.1 (1.6)
Breaking time (s) (with conversation)	1.2 (0.3)	1.1 (0.2)	1.2 (0.3)	1.1 (0.3)	0.8 (0.1)	1.0 (0.2)
Breaking time (s) (without conversation)	1.2 (0.3)	1.1 (0.2)	1.1 (0.3)	1.0 (0.2)	0.8 (0.1)	0.9 (0.2)

M, Mean; SD, Standard Deviation; MMSE, Mini-Mental State Examination.

**Fig. 1:**
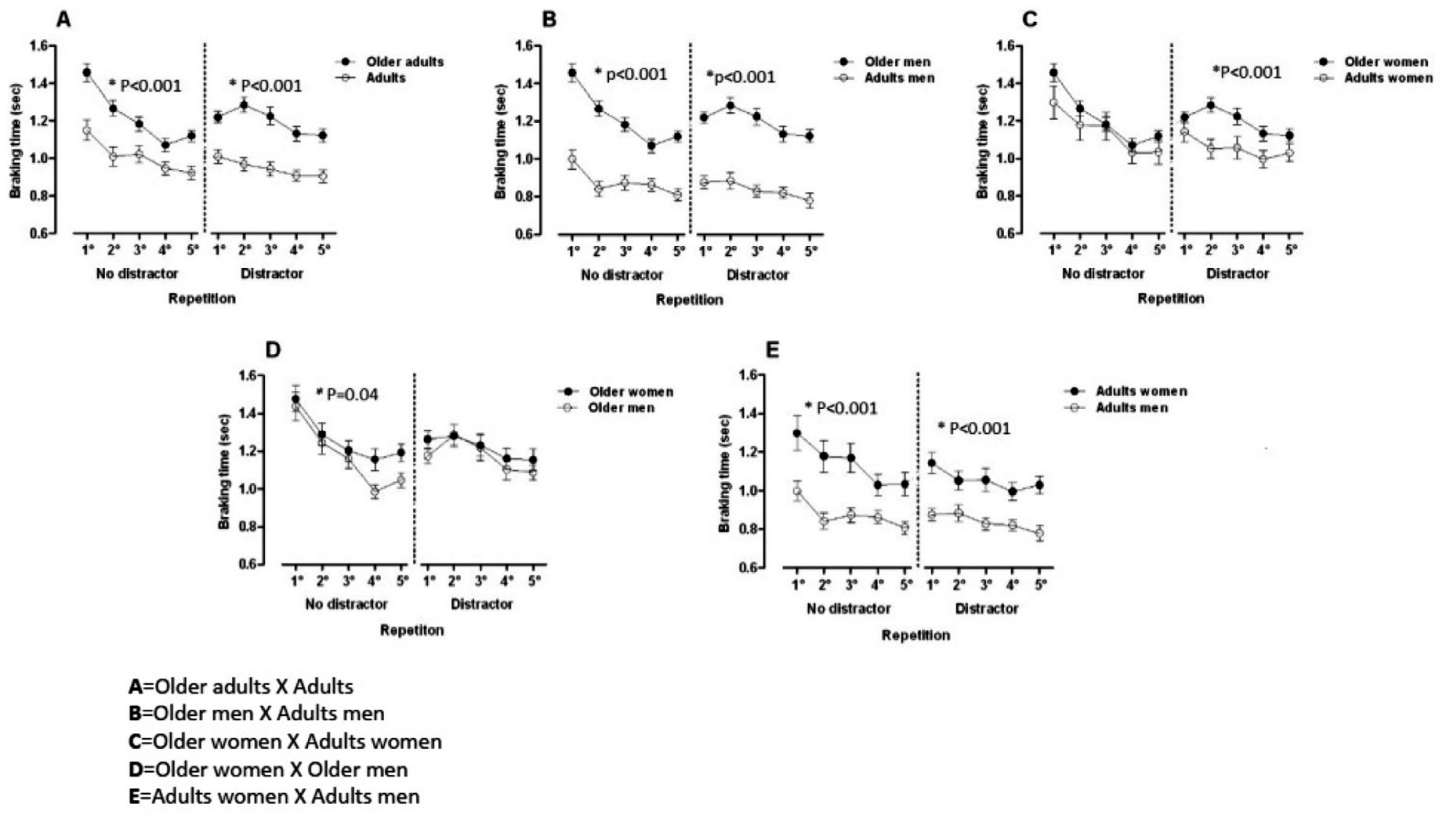
Different groups (age and genre) comparing breaking time with and without distraction.

During the driving task without the distractor, adaptation during the repetitions (tests 1 to 5) (F1.4 = 15.3; *p* < 0.001) was affected by the groups (F1.3 = 35.1; *p* < 0.001), but not by the interaction (repetitions * groups) (F1.19 = 0.9, *p* = 0.53). Multiple comparisons with the Bonferroni test showed that the older women (Test 1 and 3, *p* = 0.01) and the older men (Test 1 and 3, *p* = 0.004) adapted to the simulator after the third attempt. The middle-aged adult women maintained a constant value between the tests without significant differences, thus there was no adaptation process, and the middle-aged adult men adapted in the second repetition (Test 1 and 2; *p* = 0.05) (Fig. 1).

Comparing the groups by age and sex (without distractor) was observed that there were significant differences between the older women and older men (*p* = 0.04); middle-aged adult women and men (*p* = 0 < 0.001) and middle-aged adult men and older men (*p* < 0.001); older adults and middle-aged adults (*p* < 0.001) and middle-age adult women and middle-age adult men (*p* < 0.001) in the adaptation time (Fig. 1).

Still in the driving task in the presence of the distractor, the adaptation during the repetitions (Tests 1 to 5) (F(4) = 4.546, *p* = 0.001) was affected by the group (F(3) = 44.775, *p* < 0.001). The interaction between group*repetitions was significant (F(19) = 7121.8, *p* < 0.001) (Fig. 1).

The older women didn't present adaptations during the five repetitions (F(4) = 0.829, p=0.508) as well as middle-aged adult women (F(4) = 0.604, *p* = 0.661) and middle-aged adult men (F(4) = 1.263, *p* = 0.287). The older men adjusted in the last test (*p* = 0.057) (Fig. 1).

Comparing the groups by age and sex (with distractor) the authors observed that there were significant differences between the older women and middle-aged adult women and middle-aged adult men and older men (*p* < 0.001); older men and middle-aged adult women and middle-aged adult and older adults (*p* < 0.001) and between middle-age adult women and men (*p* < 0.001) in the time of adaptation (Fig. 1).

The linear regression model for the analysis of braking time as a dependent variable in older adults is shown in Table 2. In model 1, the independent variables age and the number of years licensed explain 16% of the braking time in the non-distractor situation and 23% with the distractor. In model 2, the independent variables age, the number of years licensed, and cognitive domain explains 17% of braking time without the distractor and 23% with the distractor. In model 3, the independent variables age, number of years licensed, cognitive domain, and motor explain 38% of braking time without the distractor and 35% with the distractor.

**Table 2:** Linear regression model using braking time with and without distraction as a dependent variable in the older women (*n* = 51).

Variables	Model 1		Model 2		Model 3	
	β	SE	β	SE	β	SE
Without distraction						
Socio-demographic domain						
Age (years)	0.411	0.011	0.439	0.011	0.249	0.011
Years od licensed (years)	-0.023	0.006	-0.026	0.006	0.060	0.006
Cognitive domain						
MMSE			-0.011	0.020	-0.080	0.021
Years of education (years)			0.116	0.016	0.170	0.018
Motor Domain						
Hand grip (kgf) (DS)					-0.259	0.012
Hand grip(kgf) (NDS)					0.067	0.017
PT/BW (%)					-0.383	0.003
Total work (J)					0.359	0.002
TUGT (s)					-0.102	0.040
Cognitive TUGT (s)					0.390	0.024
R square	0.16	0.17	0.38			
With distraction						
Socio-demographic domain						
Age (years)	0.481	0.012	.480	.013	.346	.014
Years od licensed (years)	-0.004	0.007	-0.003	0.007	0.046	0.008
Cognitive domain						
MMSE			-0.008	0.023	0.066	0.025
Years of education (years)			0.003	0.018	0.008	0.021
Motor Domain						
Hand grip (kgf) (DS)					-0.058	0.015
Hand grip(kgf) (NDS)					-0.194	0.020
PT/BW (%)					-0.174	0.004
Total work (J)					0.173	0.002
TUGT (s)					-0.051	0.048
Cognitive TUGT (s)					0.246	0.028
R square	0.23	0.23	0.35			

β, Beta Value; SE, Standard Error; MMSE, Mini Mental State Examination; kgf, Kilogram-force; DS, Dominant Side; NDS, Non-Dominant Side; PT/BW, Peak Torque corrected for Body Weight of the ankle's plantar flexors; J, Joules; TUGT, Time Up Go Test; s, seconds.

Table 3 shows the linear regression model for analysis of braking time as a dependent variable in the older adults group. In model 1, the independent age and number of years licensed variables to explain 1% of the braking time in the non-distractor situation and 11% with the distractor. In model 2, the independent variables age, the number of years licensed, and the cognitive domain explains 18% of braking time without the distractor and 11% with the distractor. In model 3, the independent variables age, number of years licensed, cognitive domain, and motor explain 12% of braking time without the distractor and 21% with the distractor.

**Table 3:** Linear regression model using braking time with and without distraction as a dependent variable in the older men (*n* = 51).

Variables’	Model 1		Model 2		Model 3	
	*β*	SE	β		β	SE
Without distraction						
**Socio-demographic domain**						
Age (years)	-0.064	0.008	-0.080	0.008	0.031	0.010
Years od licensed (years)	-0.082	0.005	-0.67	0.005	-0.107	0.006
**Cognitive domain**						
MMSE			-0.006	0.017	-0.007	0.021
Years of education (years)			-0.051	0.013	-0.086	0.014
**Motor Domain**						
Hand grip (kgf) (DS)					-0.132	-0.008
Hand grip(kgf) (NDS)					0.325	0.009
PT/BW (%)					0.391	0.002
Total work (J)					-0.382	0.001
TUGT (s)					-0.032	0.039
Cognitive TUGT (s)					0.124	0.031
**R square**	**0.01**	**0.18**	**0.12**			
With distraction						
**Socio-demographic domain**						
Age (years)	0.252	0.007	0.221	0.008	0.073	0.009
Years od licensed (years)	0.125	0.005	0.155	0.005	0.203	0.005
**Cognitive domain**						
MMSE			-0.022	0.015	-0.055	0.018
Years of education (years)			-0.101	0.011	-0.171	0.012
**Motor Domain**						
Hand grip (kgf) (DS)					-0.409	0.007
Hand grip(kgf) (NDS)					0.124	0.008
PT/BW (%)					0.048	0.002
Total work (J)					-0.005	0.001
TUGT (s)					-0.017	0.035
Cognitive TUGT (s)					-0.054	0.028
**R square**	**0.11**	**0.11**	**0.21**			

β, Beta Value; SE, Standard Error; MMSE, Mini-Mental State Examination; kgf, Kilogram-force; DS, Dominant Side; NDS, Non-Dominant Side; PT/BW, Peak Torque corrected for Body Weight of the ankle's plantar flexors; J, Joules; TUGT, Time Up Go Test; s, seconds.

Table 4 shows the predictors of braking time with and without the distractor in older adult drivers divided by sex. The cognitive TUGT explained 18% of braking time in older adults without the distractor. The age and hand grip strength of the non-dominant side together explained 30% of the braking time of the distracted older women. In the older men, the hand grip strength of the dominant side explained 8% of braking time with the distractor.

**Table 4:** Multiple Stepwise Linear Regression for predictors of braking time with and without distractor in the older adults divided by sex.

Older women	*β*	SE	p	r^2^Adjusted
Without distractor				
Cognitive TUGT (s)	0.428	0.013	0.002	0.18
With distractor				
Age (Years)	0.433	0.10	0.001	0.30
Hand grip (kgf) (NDS)	-0.05	0.002	0.008	
Older men				
With distractor				
Hand grip (kgf) (DS)	-0.294	0.004	0.036	0.08

β, Beta Value; SE, Standard Error; MMSE, Mini-Mental State Examination; kgf, Kilogram-force; DS, Dominant Side; NDS, Non-Dominant Side; PT/BW, Peak Torque corrected for Body Weight of the ankle's plantar flexors; J, Joules; TUGT, Time Up Go Test; s, seconds.

## Discussion

Age, sex, and the presence of a distractor interfere with the adaptability to perform the critical tasks of driving in the driving simulator, such as braking. In older women, TUG with distraction (dual task: cognitive and motor), age, and muscle strength were the most determinant factors in the braking time, a distinct behavior of the older adults, in which only muscle strength was identified as a factor.

Older women and older men required more attempts to adapt to the driving simulator than middle-aged adults men. These results are similar to those of Kawano et al.^12^ who report that these differences occur because middle-aged adults are more adapted to virtual environments like in daily activities. Ball et al.^13^ reported that older adults have a deficiency in divided and selective attention and the ability to rapidly process visual information, factors that may justify the longer adaptation time. The need for an adaptation period in a virtual environment of the driving simulator can be transferred, for example, to driving different vehicle models. Thus, a recommendation, particularly for older drivers, would be to avoid driving unfamiliar vehicles in the most dangerous conditions (at nighttime, in the rain, and on high-speed highways).

Women (older adults and middle-aged adults) require more attempts to adapt to the driving simulator, which is different from the results of Sahami and Sayed,^7^ who did not find differences between the sexes concerning the average speed adaptation, although a smaller sample size of 24 subjects was evaluated in this study.

A simple conversation (distractor) interfered with the ability to adapt to the driving simulator. No similar studies were found for comparison; however, it is an important finding because conversations with other occupants in a vehicle are common in actual driving environments, and can, therefore, cause accidents. Strayeret et al.^14^ attribute these deficiencies to the cognitive domain by diverting attention during the processing of information necessary to drive safely the vehicle.

The multifactorial model, including demographic, motor, and cognitive, was able to explain 38% (without distractors) and 35% (with distractors) in older women and 12% (without distractors) and 21% (with distractors) in the older men, in the braking time, which's a key action for safe driving. The distractor factor acts on motor and cognitive responses and needs to be considered in the development of interventions to improve vehicular operating capacity in older adults. Anstey et al.^15^ and Alonso et al.^11^ showed that cognitive and physical factors are related to safe driving. Safe driving requires an accurate assessment of each situation, rapid processing of information for decision-making, and fast response.

The regression model showed that cognitive TUG performance explained 18% of braking time in older women. Asimakopulos et al.^16^ report that the adequate execution of the dual task (driving) associating the breaking function with motor responses is fundamental for safe driving: to understand what happens in the environment and to respond at an appropriate time.

In older women, age and muscle strength explained 30% of the braking time, in the presence of the distractor. Awadzi et al.^17^ demonstrated that women have a greater risk of traffic accidents due to lower muscle strength. Dykiert et al.^18^ states that the action of estrogen on the cingulate cortex can affect the attention system of women and impair driving performance. Another important socio-cultural factor is the lower experience and frequency with which women drive in comparison to men in the same family.^19^

Some of the findings of this study, which relate the functional and cognitive losses of aging to the changes in driving, were already known, and there is a vast amount of literature on this subject, which is certainly very present due to the aging of populations.^11^^,^^20^ However, the question of adapting or learning new tasks involving the integration of motor and cognitive activity with quick decision-making, such as driving, can be hampered by distractions such as conversation, age, and sex. For older adults, some usual occurrences such as using a new vehicle, driving in unfamiliar places, or using guidance technologies can act as distractors (double task) and can decrease safety.

Age and sex interfere in the period of adaptation of the individual to the new task (driving in a simulator). Driving simulator studies require a period of adaptation so that the evaluation is closer to actual driving conditions.^7^ The need for a longer adaptation period is not restrictive for driving, but the drivers must know their limits, thus ensuring greater safety.

Requirements for older drivers for vehicular operating capacity would need to be broader, as the functional and cognitive losses of aging are insidious and affect people in different ways.^11^

Some study limitations are related to the multifactorial ability necessary for driving. Some other variables, not addressed in the present research, such as vision and more specific cognition, may also interfere. The driving simulator does not accurately reflect real-life situations, but it is very useful for assessing the skills required to drive, for safety, control, and standardization of the tests performed.

## Conclusion

This study showed that cognitive and physical factors are related to safe driving, and functional and cognitive losses of aging to the changes in driving.

An important contribution of this study is that drivers' companions should avoid distracting them, even with a simple conversation, as this reduces vehicle driving safety.

In addition, maintaining physical activity and/or physical exercise, prioritizing muscle strength, is an important task to keep these elderly people driving longer, as well as to work on their cognitive functions.

## Funding

This study was financed by the Fundação de Amparo à Pesquisa (Foundation for Research Support) process n° 2012/20627-5 and A scientific Cooperation agreement between Fundação de Amparo à Pesquisa and University of Michigan, process n° 13/50138-9.

## Authors' contributions

Alexandra Carolina Canonica: Conceptualization, Formal analysis, Investigation, Resources, Writing – original draft.

Angelica Castilho Alonso: Conceptualization, Formal analysis, Methodology, Resources, Writing – review & editing.

Guilherme Carlos Brech: Conceptualization, Formal analysis, Writing – review & editing.

Mark Peterson: Writing – original draft, Writing – review & editing.

Natália Mariana Silva Luna: Formal analysis, Resources, Writing & original draft.

Alexandre Leopold Busse: Formal analysis, Resources, Writing – original draft.

Wilson Jacob-Filho: Writing-review & editing.

Juliana Leme Rosa: Formal analysis, Writing – review & editing.

Jose Maria Soares-Junior: Formal analysis, Writing – review & editing.

Edmund Chada Baracat: Formal analysis, Writing – review & editing.

Júlia Maria D'Andrea Greve: Funding aquisition, Project administration, Resources, Supervision, Writing – review & editing.

## Conflicts of interest

The authors declare no conflicts of interest.

## References

[1] Wood JM, Anstey KJ, Kerr GK, Lacherez PF, Lord S. (2008). A multidomain approach for predicting older driver safety under in-traffic road conditions. J Am Geriatr Soc.

[2] Tefft BC (2014). Driver license renewal policies and fatal crash involvement rates of older drivers, United States, 1986–2011. Inj Epidemiol.

[3] Hancock PA, Lesch M, Simmons L (2003). The distraction effects of phone use during a crucial driving maneuver. Accid Anal Prev.

[4] Horrey WJ, Lesch MF, Garabet A (2008). Assessing the awareness of performance decrements in distracted drivers. Accid Anal Prev.

[5] Horrey WJ, Lesch MF (2009). Driver-initiated distractions: examining strategic adaptation for in-vehicle task initiation. Accid Anal Prev.

[6] Liu Y-C, Ou Y-K (2011). Effects of age and the use of hands-free cellular phones on driving behavior and task performance. Traffic Inj Prev.

[7] Sahami S, Sayed T (2010). Insight into steering adaptation patterns in a driving simulator. Transp Res Rec J Transp Res Board.

[8] Lourenço RA; Veras RP. Mini-Exame do Estado Mental: características psicométricas em idosos ambulatoriais Mini-Mental State Examination: psychometric characteristics in elderly outpatients 2006;40(4):712-9. 10.1590/s0034-89102006000500023 16906312

[9] Alonso C, Nata I, Luna M, Dionı IFN, Andre MD (2014). Functional Balance Assessment : review.

[10] Brech GC, Alonso AC, Luna NMS, Greve JM (2013). Correlation of postural balance and knee muscle strength in the sit-to-stand test among women with and without postmenopausal osteoporosis. Osteoporos Int.

[11] Alonso AC, Peterson MD, Busse AL, Jacob-Filho W, Borges MTA, Serra MM (2016). Muscle strength, postural balance, and cognition are associated with braking time during driving in older adults. Exp Gerontol.

[12] Kawano N, Iwamoto K, Ebe K, Aleksic B, Noda A, Umegaki H (2012). Slower adaptation to driving simulator and simulator sickness in older adults. Aging Clin Exp Res.

[13] Ball K, Owsley C, Sloane ME, Roenker DL, Bruni JR (1993). Visual attention problems as a predictor of vehicle crashes in older drivers. Invest Ophthalmol Vis Sci.

[14] Strayer DL, Drews FA (2004). Profiles in driver distraction: effects of cell phone conversations on younger and older drivers. Hum Factors.

[15] Anstey KJ, Wood J, Lord S, Walker JG (2005). Cognitive, sensory and physical factors enabling driving safety in older adults. Clin Psychol Rev.

[16] Asimakopulos J, Boychuck Z, Sondergaard D, Poulin V, Ménard I, Korner-Bitensky N (2012). Assessing executive function in relation to fitness to drive: a review of tools and their ability to predict safe driving. Aust Occup Ther J.

[17] Awadzi KD, Classen S, Hall A, Duncan RP, Garvan CW (2008). Predictors of injury among younger and older adults in fatal motor vehicle crashes. Accid Anal Prev.

[18] Dykiert D, Der G, Starr JM, Deary IJ (2012). Sex differences in reaction time mean and intraindividual variability across the life span. Dev Psychol.

[19] Classen S, Shechtman O, Awadzi KD, Joo Y, Lanford DN (2010). Traffic violations versus driving errors of older adults: informing clinical practice. Am J Occup Ther.

[20] Lacherez P, Wood JM, Anstey KJ, Lord SR (2014). Sensorimotor and postural control factors associated with driving safety in a community-dwelling older driver population. J Gerontol A Biol Sci Med Sci.

